# Symbiotic incompatibility between soybean and *Bradyrhizobium* arises from one amino acid determinant in soybean Rj2 protein

**DOI:** 10.1371/journal.pone.0222469

**Published:** 2019-09-13

**Authors:** Masayuki Sugawara, Yosuke Umehara, Akito Kaga, Masaki Hayashi, Masao Ishimoto, Shusei Sato, Hisayuki Mitsui, Kiwamu Minamisawa

**Affiliations:** 1 Graduate School of Life Sciences, Tohoku University, Sendai, Miyagi, Japan; 2 Institute of Agrobiological Sciences, National Agriculture and Food Research Organization, Tsukuba, Ibaraki, Japan; 3 National Institute of Crop Science, National Agriculture and Food Research Organization, Tsukuba, Ibaraki, Japan; Huazhong Agriculture University, CHINA

## Abstract

Cultivated soybean (*Glycine max*) carrying the *Rj2* allele restricts nodulation with specific *Bradyrhizobium* strains via host immunity, mediated by rhizobial type III secretory protein NopP and the host resistance protein Rj2. Here we found that the single isoleucine residue I490 in Rj2 is required for induction of symbiotic incompatibility. Furthermore, we investigated the geographical distribution of the *Rj2*-genotype soybean in a large set of germplasm by single nucleotide polymorphism (SNP) genotyping using a SNP marker for I490. By allelic comparison of 79 accessions in the Japanese soybean mini-core collection, we suggest substitution of a single amino acid residue (R490 to I490) in Rj2 induces symbiotic incompatibility with *Bradyrhizobium diazoefficiens* USDA 122. The importance of I490 was verified by complementation of *rj2-*soybean by the dominant allele encoding the Rj2 protein containing I490 residue. The *Rj2* allele was also found in *Glycine soja*, the wild progenitor of *G*. *max*, and their single amino acid polymorphisms were associated with the *Rj2*-nodulation phenotype. By SNP genotyping against 1583 soybean accessions, we detected the *Rj2*-genotype in 5.4% of *G*. *max* and 7.7% of *G*. *soja* accessions. Distribution of the *Rj2*-genotype soybean plants was relatively concentrated in the temperate Asian region. These results provide important information about the mechanism of host genotype-specific symbiotic incompatibility mediated by host immunity and suggest that the *Rj2* gene has been maintained by environmental conditions during the process of soybean domestication.

## Introduction

Cultivated soybean (*Glycine max* [L.] Merr.) is an important leguminous crop and source of nutrition for humans and livestock worldwide. Wild soybean (*Glycine soja* Sieb. and Zucc.) is also an important genetic resource for soybean breeding. It has been suggested that *G*. *max* was domesticated from *G*. *soja* in East Asia 6000–9000 years ago [1,2]. Soybeans are agriculturally significant plants. Therefore, the soybean whole-genome sequence [3–5], genome-wide single nucleotide polymorphism (SNP) genotype [6–8], and germplasm core collections [7,9,10] have been established. This genetic information is used to identify an agricultural important allele and track the evolutionary history of soybean domestication.

Soybean plants form root nodules upon infection with nitrogen-fixing rhizobia such as *Bradyrhizobium diazoefficiens*, *B*. *japonicum*, *B*. *elkanii*, and *Ensifer fredii* [11–13]. Nitrogen fixation efficacy of the nodules can affect plant growth and depends on the species and strains of root nodule bacteria, because symbiotic nitrogen-fixation capacity varies among soybean-nodulating strains [14,15]. However, the population structure of indigenous rhizobia in soybean nodules is influenced not only by environmental conditions (e.g. soil pH and temperature) but also by the role of soybean genes that restrict nodulation to specific rhizobia [16–20].

Several dominant alleles (*Rj2*, *Rj3*, *Rj4*, and *Rfg1*) in *G*. *max* are known to restrict nodulation to specific rhizobial strains [21]. The *Rj* genotypes, rather than the recessive *rj* genotypes, would be suitable to eliminate infection with those indigenous rhizobia in order to promote nodulation by specific inoculants with high symbiotic nitrogen-fixing ability [21,22]. Among the cultivars carrying these alleles, the *Rj2–*genotype of *G*. *max* cultivars blocks nodule induction by specific *B*. *diazoefficiens* and *B*. *japonicum* strains, including strain USDA 122 [23,24]. The *Rj2*-genotype of various soybean cultivars has been identified by a phenotyping analysis with an incompatible strain [25–27]. However, an investigation into the determinant of *Rj2*-mediated symbiotic incompatibility in soybeans is required for manipulating the genotype and efficient surveying of the *Rj2*-genotype in diverse soybean accessions by genetic analysis, such as single nucleotide polymorphism (SNP) genotyping.

Yang and colleagues [28] reported that *Rj2* encodes a typical resistance (R) protein of the Toll-interleukin receptor / nucleotide-binding site / leucine-rich repeat (TIR-NBS-LRR) class. Plant R proteins containing both NBS and LRR domains often directly or indirectly recognize pathogenic effectors and mount a strong immune response that is referred to as effector-triggered immunity (ETI) [29,30]. The inactivation of the type III protein secretion system (T3SS) in *B*. *diazoefficiens* USDA 122 restores its nodulation capability in the *Rj2* soybean cultivar ‘Hardee’ [31]. We recently revealed that variation in the T3SS-secretory protein NopP acts as a rhizobial determinant for *Rj2*-mediated symbiotic incompatibility [24]. These findings indicate that *Rj2*-soybeans monitor the specific variants of rhizobial NopP via the Rj2 protein, and thereby block infection by incompatible rhizobia through the process of ETI.

**Fig 1:**
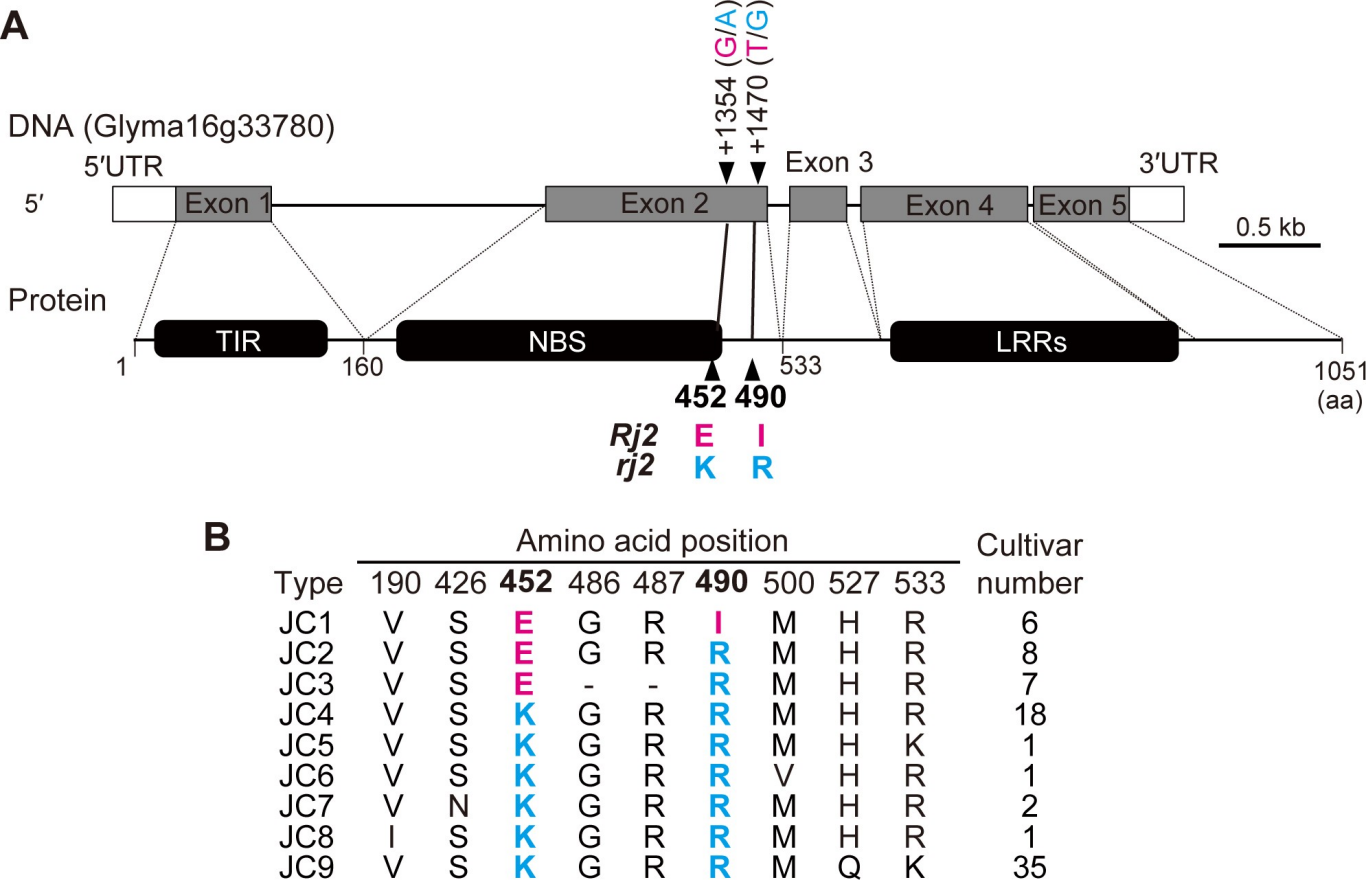
The structure of the *Rj2* gene and amino acid polymorphisms in a Japanese soybean mini-core collection. (A) Structure of the *rj2* allele (Glyma16g33780) in the model soybean ‘Williams 82’. The gene consists of five exons (Exon 1 to Exon 5), and encodes a TIR-NBS-LRR class resistance protein. The residues at positions 452 and 490 are encoded by Exon 2. Numbers and nucleotides following the arrowhead on Exon 2 indicate the SNP position on the *rj2* cDNA (Accession No. GU 967692) and also indicate the nucleic acids involved in the differences in amino acid residues at 452 or 490. The *Rj2*- and *rj2*-type amino acids or nucleotides are shown in magenta and cyan letters, respectively. (B) Allelic comparison of the 79 soybean accessions in the Japanese mini-core collection. Amino acid sequences of each cultivar were deduced from the Exon 2 region in the alleles.

An allelic comparison of 21 cultivars of *G*. *max* revealed that the *Rj2* alleles translate a protein containing residues of glutamic acid (E) and isoleucine (I) at positions 452 and 490, respectively, whereas the products of the recessive allele *rj2* share a haplotype containing residues of lysine (K) and arginine (R) at the same positions as E and I, respectively [27] (Fig 1A). Furthermore, an *rj2*-soybean complemented with the chimeric *Rj2* gene, which translates a protein having E452K and I490R substitutions in the *Rj2* allelic product, did not induce NopP-mediated incompatibility with *B*. *diazoefficiens* USDA 122 [24].

Although previous studies have considered the mechanisms of *Rj2*-mediated incompatibility with bradyrhizobia, the specific amino-acid residue(s) in the Rj2 protein responsible for this incompatibility have not been identified. Moreover, the evolutionary process behind this symbiotic compatibility in soybean-bradyrhizobium interactions remains unclear, because the *Rj2* and *rj2* alleles in *G*. *soja* have not been identified. In this study, we report that a single isoleucine residue in Rj2 is responsible for *Rj2*-mediated symbiotic incompatibility. In addition, we identified the *Rj2* allele in *G*. *soja* accessions and investigated the geographical distribution of the *Rj2* allele in 1583 soybean accessions using SNP genotyping.

## Results

### Allelic comparison of *Rj2* in Japanese soybean mini-core collection

In the reference genomic sequence of *G*. *max* ‘Williams 82,' the recessive allele *rj2* (Glyma16g33780) encodes a 1,051 bp amino-acid protein containing TIR, NBS, and LRR domains (Fig 1A). The amino-acid residues at positions 452 and 490 are encoded by the second (Exon 2) of five exons (Fig 1A). In order to compare the amino-acid sequence of the Rj2/rj2 allelic product in soybean resources, we first determined the nucleotide sequence of the whole Exon 2 region in the genomes of soybean accessions in Japanese soybean mini-core collection [7]; in 2012, this collection consisted of 79 Japanese cultivars. Pairwise comparisons of the deduced amino-acid sequences revealed nine sequence patterns (JC1 to JC9; Fig 1B). Haplotypes containing both *Rj2*-type E452 and *Rj2*-type I490 were estimated from six cultivars (Chizuka Ibaraki 1, Date Cha Mame, Aobako, Kurakake, Maetsue Zairai 90B, and Kumaji 1) classified in JC1 (Fig 1B, S1 Table). The allelic products of 58 cultivars belonging to JC4 to JC9 contained both of the *rj2*-type residues, K452 and R490 (Fig 1B, S1 Table). In addition, an intermediate haplotype containing E452 and R490, which was not observed by Yang et al. [28], was found in the remaining 15 cultivars classified in JC2 or JC3 (Fig 1B, S1 Table).

### Nodulation phenotype of Japanese cultivars inoculated with *Bradyrhizobium diazoefficiens* USDA 122

**Fig 2:**
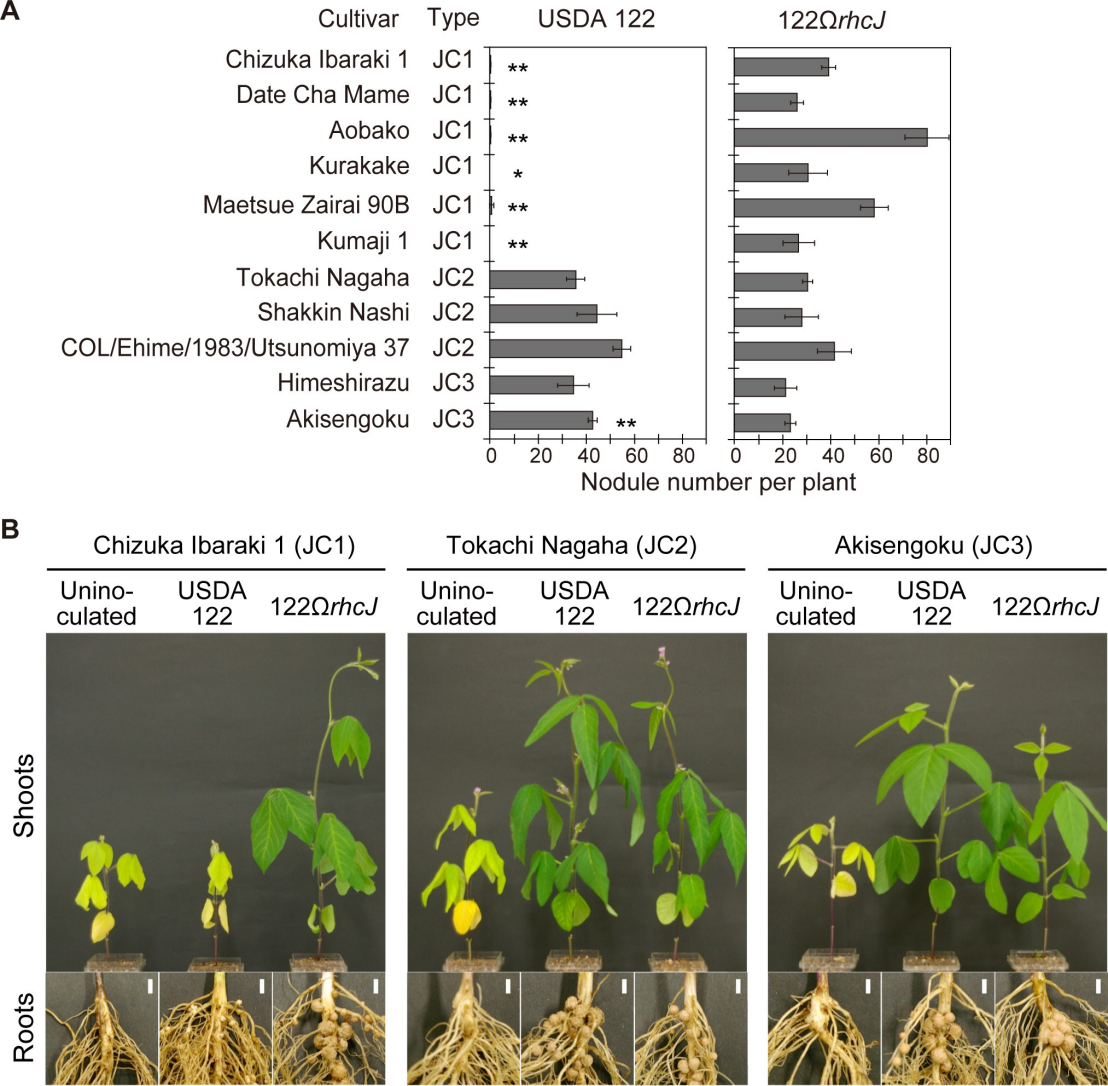
Nodulation phenotype of *Glycine max* cultivars following inoculation with *Bradyrhizobium diazoefficiens* USDA 122 and its *rhcJ* mutant. (A) Number of nodules formed on roots inoculated with USDA 122 or 122Ω*rhcJ* 28 days after inoculation. The *Rj2*-type was assigned on the basis of the deduced amino acid sequence (see S1 Table). Error bars show SEM (*n* = 3 or 4). Significant differences from 122Ω*rhcJ* were detected using Student’s *t*-test: *P < 0.05, **P < 0.01. (B) Shoots and roots of *Glycine max* cultivars Chizuka Ibaraki 1, Tokachi Nagaha, and Akisengoku. Scale bars: 5 mm.

Next, we investigated the symbiotic phenotype of soybean cultivars with sequence pattern JC1 (E452/I490) and newly identified sequence patterns JC2 and JC3 (E452/R490) with *B*. *diazoefficiens* USDA 122 and its T3SS-deficient mutant (122Ω*rhcJ*). Nodule number at 28 days after inoculation (DAI) indicated that all cultivars identified as JC1 failed to nodulate with *B*. *diazoefficiens* USDA 122 (less than 1 nodule per plant, Fig 2). The cultivars inoculated with the T3SS mutant (122Ω*rhcJ*) formed more nodules (approximately 26–80 nodules per plant) and grew well in nitrogen-free medium (Fig 2). These results clearly indicate that the six cultivars identified as JC1 were of the *Rj2*-genotype, because nodulation by *B*. *diazoefficiens* USDA 122 is restricted by T3SS, as has been observed in the *Rj2*-soybean cultivars Hardee, CNS and IAC-2 (Sugawara et al. 2018; Tsukui et al. 2013). Meanwhile, the representative JC2- and JC3-type soybean cultivars inoculated with *B*. *diazoefficiens* USDA 122 formed many nodules (approximately 35–55 per plant) and the symbiotic phenotypes were similar to those inoculated with 122Ω*rhcJ* in terms of the number of formed nodules and growth (Fig 2). Therefore, soybean cultivars having E452 and R490 in the allelic product were of the *rj2*-genotype, suggesting that symbiotic compatibility with *B*. *diazoefficiens* USDA 122 may be determined by the difference in single residue at 490 (I or R) (Fig 1).

### Determination of amino acid residue responsible for *Rj2* symbiotic compatibility

**Fig 3:**
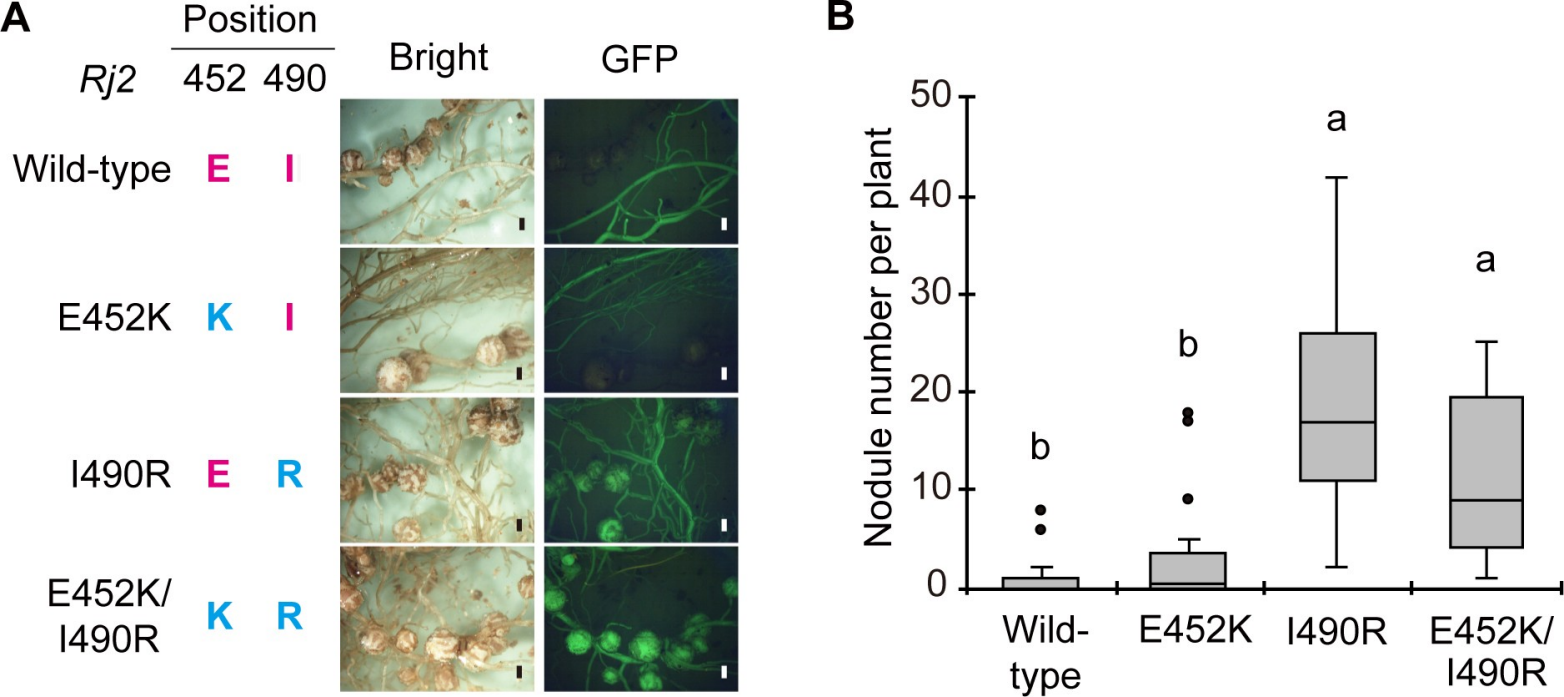
Nodulation of *rj2* soybean cultivar Lee transformed with the *Rj2* gene and its mutant allele inoculated with *Bradyrhizobium diazoefficiens* USDA 122. (A) Bright-field images and GFP fluorescence images of roots of transgenic cultivar Lee complemented with the wild-type *Rj2* of Hardee (wild type) or its mutant alleles (E452K, I490R, and E452K/I490R). *Bradyrhizobium diazoefficiens* USDA 122 was inoculated onto each seedling, and images were taken at 4 weeks after inoculation. Scale bars: 1 mm. (B) Box-and-whisker plots showing the number of nodules formed on GFP-expressing hairy roots. Center line: median; box limits: first and third quartiles; whiskers: ranges; closed circles: outliers. Wild type: *n* = 13; E452K: *n* = 16; I490R: *n* = 17; E452K/I490R: *n* = 11. Different letters above bars indicate statistical significance (*P* < 0.01; nonparametric Steel–Dwass multiple comparison test). The *Rj2*- and *rj2*-type amino acids are shown in magenta and cyan letters, respectively.

To examine whether symbiotic incompatibility with USDA 122 is determined by a single amino acid residue at position 490 in Rj2 protein, we produced transgenic *rj2-*soybean cultivar Lee complemented with *Rj2* and its chimeric genes. We prepared cDNAs of *Rj2* obtained from cultivar Hardee and its chimeric DNAs that encode the Rj2 protein containing E452K and/or I490R substitutions (E452K, I490R, and E452K/I490R), which were introduced using a binary vector, pUB-GW-GFP (S2 Table). As hairy root transformation was conducted without antibiotic selection, the resulting hairy roots were either transgenic or non-transgenic. It was possible to distinguish the transgenic and non-transgenic plants by detecting green fluorescent protein (GFP) encoded in the binary vector. Following inoculation with USDA 122, nodules were formed infrequently on transgenic hairy roots of plants containing *Rj2* wild-type cDNA and E452K cDNA (Fig 3A). In contrast, roots of plants transformed with cDNAs of I490R and E452K/I490R showed normal nodulation (Fig 3A). The number of nodules on wild-type and E452K-transgenic roots was significantly lower than those on I490R and E452K/I490R transgenic roots (Fig 3B). These results demonstrate that the substitution (R to I) of the amino acid residue at position 490 in the protein fully determines the *Rj2*-phenotype.

### SNP-based *Rj2* genotyping in cultivated soybean resources

**Fig 4:**
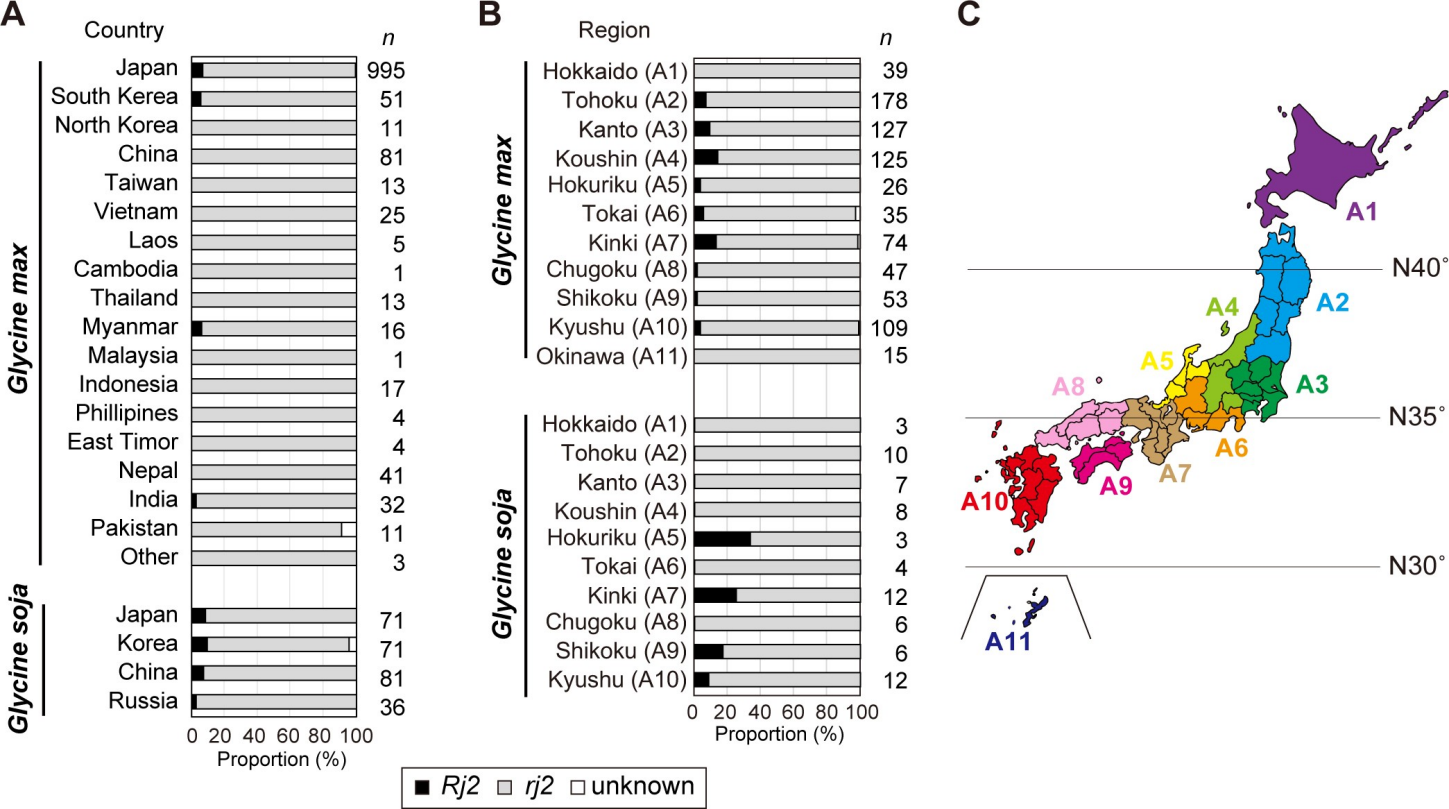
Geographical distribution of *Rj2*-genotype soybean germplasm. (A) Proportion of *Rj2* or *rj2* genotypes in soybean germplasm by country of origin. (B) Proportion of *Rj2* or *rj2* genotypes in Japanese soybean landraces by region of origin. Black and gray bars: percentage of accessions featuring *Rj2* and *rj2* genotypes, respectively. White bars: percentage of accessions that could not be determined by this analysis. *n*: numbers of tested soybean accession. (C) Map of Japan. Japan was subdivided into 11 regions (A1-A11) with reference to Kaga et al. [7]. The colors are arbitrary.

We carried out *Rj2* genotyping using an SNP marker for I490 against a large set of germplasms from *G*. *max* and *G*. *soja* accessions [7]. Among the *G*. *max* 1324 accessions, the *Rj2*-type SNP was detected in 72 accessions (5.4%), and the *rj2*-type was detected in 1248 accessions (94.3%) (Table 1). The *Rj2*-genotype *G*. *max* accessions from Japan, South Korea, Myanmar, and India, whereas the *Rj2*-genotype was not observed in accessions from the other 13 countries, including China (Fig 4A).

**Table 1:** *Rj2*-genotype in soybean germplasm detected by SNP genotyping.

	Genotype ^*a*^			Number of tested accessions
	*Rj2*	*rj2*	Unknown ^*b*^	
*Glycine max*	5.4% (72)	94.3% (1248)	0.3% (4)	1324
*Glycine soja*	7.7% (20)	91.1% (236)	1.2% (3)	259
Whole	5.8% (92)	93.7% (1484)	0.4% (7)	1583

^*a*^ Percentage and number (in parentheses) of accessions having the *Rj2* or *rj2* genotypes.

^*b*^ This includes accessions in which both *Rj2*- and *rj2*-type nucleotides were detected, and those in which neither was detected.

Because the *G*. *max* accessions tested were mainly derived from Japan (995 accessions), we analyzed the geographical distribution of the *Rj2/rj2* genotypes of *G*. *max* landraces in Japan. *Rj2*-genotype cultivated soybean was not found in the landraces that from Hokkaido (the most northern island) and Okinawa (the most southern island), but approximately 2–14% of *G*. *max* landraces originating in other areas (including mainland Japan) showed the *Rj2*-genotype (Fig 4B and 4C). In other words, *Rj2*-genotype landraces were concentrated in temperate areas in central regions in Japan, suggesting that the distribution of *Rj2* soybean is probably geographically biased.

### *Rj2* genotype and phenotype in wild soybean

Previous studies indicated that a certain line of wild soybean (*G*. *soja*) shows symbiotic incompatibility with a *Rj2*-incompatible strain [25], but the *Rj2* gene in *G*. *soja* have not been identified. According to the results of our SNP-genotyping, the *Rj2*-type SNP was detected in 20 accessions (7.7%) of the 259 *G*. *soja* accessions from Japan, Korea, China, and Russia (Table 1, Fig 4A), suggesting that *Rj2* allele similar to that in *G*. *max* may be retained in *G*. *soja*. Six of the 71 *G*. *soja* accessions (8.5%) from Japan were estimated to have the *Rj2*-genotype (Fig 4A). These wild soybeans having *Rj2*-type SNP were from the area (A5, A7, A9 and A10) which close to the latitude 35°N, and were not found in accessions originating from northern Japan or Hokkaido island (Fig 4B and 4C).

**Fig 5:**
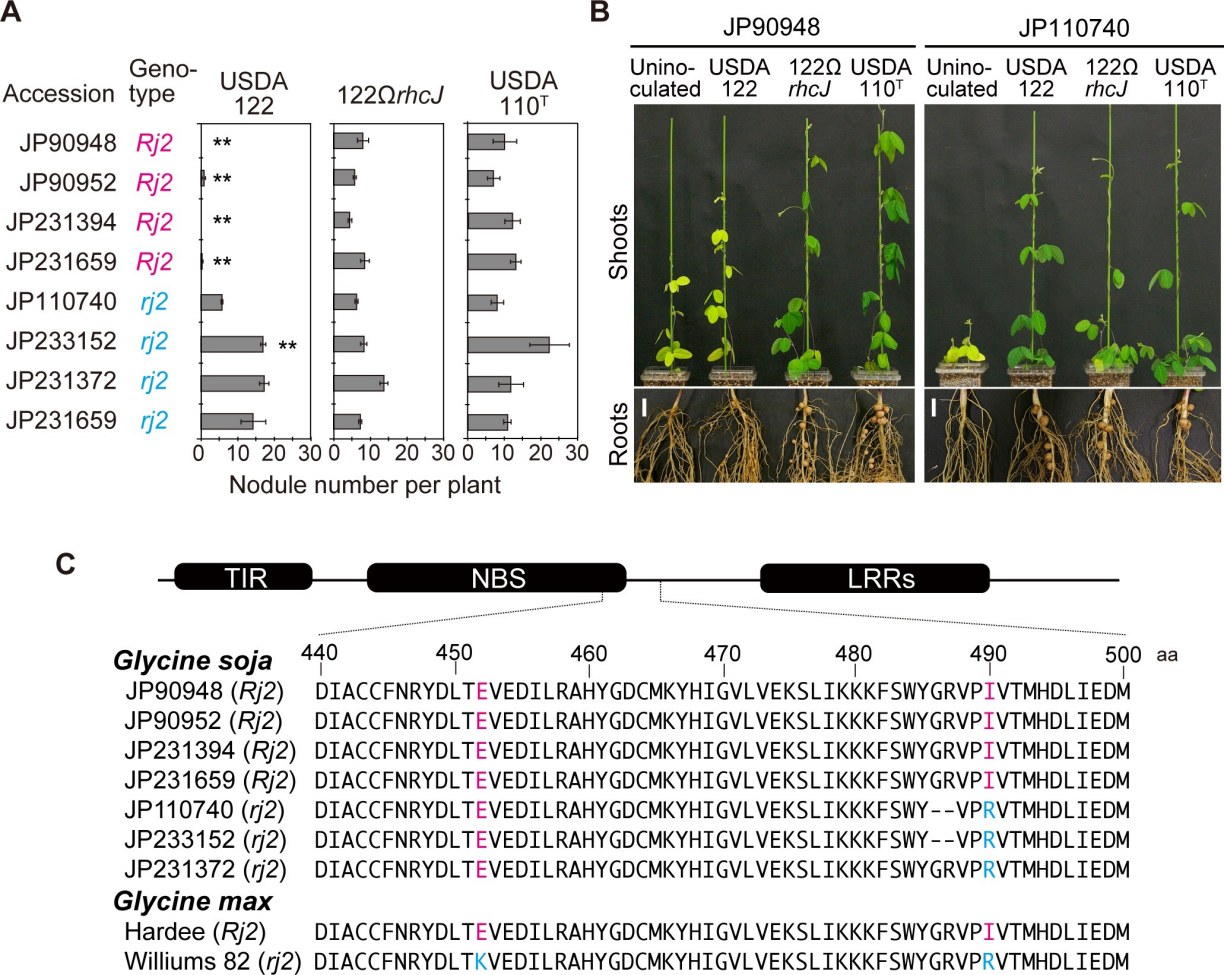
Nodulation phenotype of *Glycine soja* accessions following inoculation with *Bradyrhizobium diazoefficiens* USDA 122, 122Ω*rhcJ*, and USDA 110^T^. (A) Number of nodules formed on roots of *G*. *soja* accessions inoculated with *B*. *diazoefficiens* USDA 122, 122Ω*rhcJ*, or USDA 110^T^ 28 days after inoculation. Error bars show SEM (*n* = 4). Significant differences from 122Ω*rhcJ* were detected using Student’s *t*-test: *P < 0.05, **P < 0.01. (B) Shoots and roots of accessions JP90948 (*Rj2*) and JP110740 (*rj2*) at 28 days after inoculation with *B*. *diazoefficiens* strains. Scale bars: 1 cm. (C) Amino acid sequence alignment of the Rj2 and rj2 protein in the *G*. *soja* and *G*. *max* accessions. The *Rj2*- and *rj2*-type amino acids at positions 452 and 490 are shown in magenta and cyan letter, respectively.

To examine whether *G*. *soja* accessions with the *Rj2*-type SNP indeed show symbiotic incompatibility with *B*. *diazoefficiens* USDA 122, we inoculated *B*. *diazoefficiens* USDA 122, 122Ω*rhcJ*, and an *Rj2*-compatible strain (USDA 110^T^) on the four selected *G*. *soja* accessions having the *Rj2*- or *rj2*-genotype (S3 Table). At 28 DAI, the *G*. *soja* accessions having the *Rj2*-type SNP (JP90948, JP90952, JP231394, and JP231659) failed to form effective nodules after inoculation with *B*. *diazoefficiens* USDA 122, whereas those inoculated with 122Ω*rhcJ* formed significantly more nodules and grew well in a nitrogen-free medium (Fig 5A and 5B). The number of nodules induced by USDA 110^T^ was comparable to that induced by 122Ω*rhcJ* (Fig 5A). On the other hand, four *rj2*-type accessions (JP110740, JP233152, JP231372, and JP231484) inoculated with USDA 122 formed effective nodules as efficient as those inoculated with 122Ω*rhcJ* and USDA 110^T^ (Fig 5A and 5B).

We recently revealed that the difference of three specific amino-acid residues in a T3SS effector NopP among USDA 122 and USDA 110^T^ determines symbiotic compatibility with *Rj2*-genotype *G*. *max* [24]. To examine whether the variation of rhizobial NopP also determine symbiotic incompatibility with *Rj2*-genotype *G*. *soja*, we inoculated USDA 122 derivative carrying USDA 110-type *nopP* (122*nopP*_110_). Four accessions of *Rj2*-genotype *G*. *soja* inoculated with 122*nopP*_110_ formed significantly more nodules on the roots than did wild-type USDA 122 and the leaves of 122*nopP*_110_ were green, indicating nitrogen-fixing activity of the nodules (S1 Fig). These results suggest that induction of symbiotic incompatibility between USDA 122 and *G*. *soja* is mediated by NopP, as seen in *G*. *max*.

### *Rj2* allele in *Glycine soja*

In order to identify the *Rj2* gene in *G*. *soja*, we conducted a homology search against all annotated protein sequences of the model *G*. *soja* accession W05 [5]. BLASTX analysis of the Hardee *Rj2* cDNA sequence revealed an annotated protein (glysoja_039303 product) having high amino-acid sequence homology (94% identity) and containing *rj2*-type residues E452/R490. However, with a length of 538 aa, this protein was shorter than Rj2 of *G*. *max* ‘Hardee’ (1,052 aa) and without predicted LRR domain. Therefore, we determined the full-length cDNA sequence in the *G*. *soja* accession JP90948 (*Rj2*) by using 5′/3′ rapid amplification of cDNA ends (RACE). The deduced amino-acid sequence shows high homology with that of *G*. *max* ‘Hardee’ (98% identity, S3 Table) and was predicted the TIR, NBS, and LRR domains (Fig 5C). As observed for *G*. *max*, the allelic products of representative *Rj2*-genotype *G*. *soja* accessions contained I490, whereas those of the *rj2*-genotype *G*. *soja* accessions contained R490 (Fig 5C). These results suggest that the *Rj2* or *rj2* gene is conserved in *G*. *soja*, and that the *G*. *soja Rj2*-genotype restricts nodulation with some bradyrhizobial strains via the same resistance protein in *G*. *max*.

## Discussion

Genotype-specific compatibilities in plant-pathogen and plant-mutualist interactions are often determined by host immune responses, starting with the recognition of a corresponding bacterial or fungal effector protein by plant NBS-LRR protein [24,30,32]. Resistance (*R*-) genes encoding the NBS-LRR protein are abundant in every plant species and the structures are highly diverse [33–35], which provide resistance to a variety of pathogens. Yang et al. [28] predicted the functional requirement of *Rj2* for a haplotype containing both E452 and I490 in the TIR-NBS-LRR class of the Rj2 protein, following allelic comparison of 21 soybeans including Hardee (*Rj2*) and Williams 82 (*rj2*). Based on allelic comparisons using the soybean mini-core collection [7], we demonstrated that the single amino acid residue I490 in the Rj2 protein is the host determinant that induces symbiotic incompatibility with *B*. *diazoefficiens* USDA 122 (Figs 1–3). On the symbiont side, three amino acid residues (R60, R67, H173) in the NopP effector secreted via T3SS in *B*. *diazoefficiens* USDA 122 are required for symbiotic incompatibility with *Rj2*-genotype soybeans [24]. In other words, *Rj2* incompatibility depends on only a single amino acid residue in the host Rj2 protein, and three amino acid residues in rhizobial NopP proteins, providing a “lock and key” mechanism. This type of structural and functional relationship has not previously been fully observed in plant-pathogen interactions, although plant NBS-LRRs are known to detect specific pathogen effectors; they do this via diverse mechanisms including effector-mediated modifications of guardee or decoy proteins that have different evolutionary constraints [30,32]. The recognition mechanism of NopP by Rj2 protein and the role of I490 in Rj2 for activation of immune response are still unknown. Since the 490th amino acid residue (I or R) in Rj2/rj2 protein is located between NBS and LRR domains, it is considered that the change of this residue does not affect the essential function of R protein. We assume that Rj2 with uncharged isoleucine residue at position 490 and rj2 with negatively charged arginine probably have slightly different protein conformations at inactive state, and that only in the case of I490, USDA122-type NopP can specifically bind and induce immune response. Thus, future research about how the soybean Rj2 protein detects bradyrhizobial NopP variants will provide a better understanding of NBS-LRRs recognition of symbionts and pathogens by plants.

It has been suggested that cultivated soybean has lost many alleles and that genetic diversity has been eroded in the course of selection by domestication [36,37]. By cDNA sequencing and symbiotic phenotyping with *B*. *diazoefficiens* USDA 122, we found that the *Rj2* allele occurred in *G*. *soja* accessions, and that the *Rj2*/*rj2* polymorphism due to amino acid variation in *G*. *max* also occurred in *G*. *soja* (Fig 5). *Glycine soja* has 200 more *R*-genes than *G*. *max*, indicating that many *R*-genes may have been lost during the soybean domestication process [37,38]. It has also been suggested that *R*-genes underwent rapid gain-and-loss events during plant evolution to adapt to their corresponding pathogens in specific environments [38,39]. Together with our results, these findings suggest that the *Rj2* gene has been maintained during the process of soybean domestication to adapt to high rhizobial diversity.

The single amino acid residue that determines the *Rj2* and *rj2* genotypes is due to a single nucleotide base difference (Fig 1A); this enabled us to distinguish the genotypes in each soybean cultivar, using an efficient SNP-based analysis. Using SNP-genotyping targeting the SNP, we detected the *Rj2*-genotype in 5.4% of *G*. *max* and 7.7% of *G*. *soja* accessions from the 1583 accessions in the National Agricultural and Food Research Organization (NARO) Genebank (Table 1). Devine [25] previously reported that 1.8% of *G*. *max* and 12.1% *G*. *soja* accessions originating from Asian countries show symbiotic incompatibility with *B*. *japonicum* USDA 7, an *Rj2*-incompatible strain. The results of our SNP genotyping (Table 1) were similar to those from previous phenotypic analysis, in that the occurrence frequency of the *Rj2*-genotype, and the proportion of *Rj2*-genotypes among the soybean accessions were higher in *G*. *soja* than in *G*. *max*.

The *Rj2*-genotypes of *G*. *max* and *G*. *soja* were relatively more frequent among the accessions from Japan and Korea than among those from other Asian countries (Fig 4A). However, the *Rj2*-type SNP was not detected in *G*. *max* accessions from many Southeast Asian countries (Vietnam, Laos, Cambodia, Thailand, Malaysia, Indonesia, Philippines and East Timor) in the present study (Fig 4A). These results suggest that the *Rj2*-genotype soybeans are distributed mainly in the temperate Asian areas. Interestingly, the *Rj2*-genotype *G*. *max* did not occur among the 81 accessions from China, although the *Rj2*-genotype *G*. *soja* did occur in those accessions. This suggests that Chinese-cultivated soybean may have been preferentially selected for the *rj2*-genotype in the process of breeding. However, previous phenotypic analysis revealed that the *Rj2*-genotype *G*. *max* lines are concentrated in the eastern coastal region of China [26].

In Japan, the *Rj2*-genotypes of *G*. *max* and *G*. *soja* were detected in the accessions from the main island, which is in the temperate area ca. 30−40 degrees north of Japan. *Bradyrhizobium diazoefficiens* and *B*. *japonicum* dominate in soils of Japanese temperate regions as soybean symbionts [40]; the distribution of the *Rj2* soybean genotype is thus likely to be consistent with that of their incompatible rhizobia. In contrast, *Rj4*-genotype soybeans, which are restricted to nodulation involving mainly *B*. *elkanii* strains [41,42], are distributed more widely in tropical southeast Asian countries than in temperate countries [26]. *Bradyrhizobium elkanii* strains have been isolated from nodules of *G*. *max* and *G*. *soja* mainly in the subtropical and tropical regions around the world [43–45], and *Rj4* incompatible *B*. *elkanii* strains have been isolated from *G*. *max* grown in tropical countries [46,47]. These circumstances suggest that environmental conditions (e.g. temperature) and indigenous rhizobial populations may be closely related to the distribution and maintenance of *Rj* genotypes.

In conclusion, we found an amino-acid determinant of the *Rj2*-genotype in cultivated and wild soybeans. This indicates that the *Rj2* allele has been maintained in the genome during soybean domestication, suggesting that a selective advantage of *Rj2* under certain environmental conditions. These findings will contribute to better-informed management strategies for soybean production utilizing their ability to enter symbiosis with nitrogen-fixing bacteria. Additionally, our findings further increase our understanding of effector recognition mechanisms and the evolution of host genotype-specific compatibility in plant–microbe interactions.

## Materials and methods

### Plant materials

The *G*. *max* and *G*. *soja* accessions used for genotyping and phenotyping are listed in S1 and S4 Tables, respectively. All of the seeds of soybean accessions were obtained from the NARO Genebank (https://www.gene.affrc.go.jp/index_en.php).

### Bacterial strains and growth conditions

Bacterial strains and plasmids used in this study are listed in S2 Table. *Bradyrhizobium* strains were grown aerobically at 30°C in HM salt medium [48] supplemented with 0.1% (w/v) arabinose and 0.025% (w/v) yeast extract. *Escherichia coli* strains were grown at 37°C in Luria–Bertani medium [49]. *Agrobacterium rhizogenes* was grown at 28°C in Yeast Extract Peptone (YEP) medium [50]. The following antibiotics were added when needed at the indicated concentrations: for *E*. *coli*, kanamycin (Km) at 50 mg l^−1^; for *A*. *rhizogenes*, chloramphenicol at 30 mg l^−1^ or Km at 100 mg l^−1^.

### DNA sequencing of Glyma16g33780 locus in soybean mini-core collection

The genomic region of the second exon in the Glyma16g33780 locus of each soybean accession was obtained by PCR using PrimeSTAR Max DNA Polymerase (Takara Bio Inc., Kusatsu, Japan) and oligonucleotide primers Glyma_Rgene_F2 and Glyma_Rgene_R2 (S5 Table). Total DNA for the template was extracted from soybean seed using the DNeasy Plant Mini Kit (QIAGEN Inc., Hilden, Germany). The PCR product (1.5 kb) was purified with Agencourt AMPure XP (Beckman Coulter Inc., Brea, CA, USA). The nucleotide sequence of the fragment was determined using a 3730xl DNA Analyzer with a BigDye Terminator Cycle Sequencing Reaction Kit (Thermo Fisher Scientific, Inc., Waltham, MA, USA), and the primers referred to earlier. The sequences obtained were assembled, and the amino acid sequences were deduced using Genetyx-MAC v. 18.0.3 software (Genetyx Co., Tokyo, Japan).

### Plant growth conditions and nodulation assays

Seeds of *G*. *max* were surface-sterilized in 0.5% sodium hypochlorite for 1 min and then washed 10 times with sterile distilled water. Four seeds were sown in a 300 ml plant box (CUL-JAR300; Iwaki, Tokyo, Japan) containing sterile vermiculite, and were watered with a nitrogen-free plant nutrient solution [51]. *Glycine soja* seeds were scarified and surface-sterilized using concentrated sulfuric acid for 7 min. After washing, seeds were soaked in sterile distilled water and stored at 4°C overnight, and then sown as described above. *Bradyrhizobium* strains were cultured in liquid medium for 5 or 6 days. The number of cells was adjusted to 10^7^ ml^−1^ in sterile water by direct counting using a Thoma hemocytometer (Kayagaki Irika Kogyo Co. Ltd., Tokyo, Japan). Aliquots of 1 ml of the cell suspension were inoculated onto surface-sterilized seeds of *G*. *max* or *G*. *soja*. Plants were grown in a plant growth cabinet (NK Systems Co. Ltd., Osaka, Japan) at 25°C with a photoperiod of 16 h light / 8 h dark. The seedlings were thinned out to one (for *G*. *max*) or two (for *G*. *soja*) per box 7 days after sowing. The number of nodules and the dry weights of nodule and plant were determined 28 DAI.

### *Rj2* complementation analysis using hairy root transformation

Complementation of *Rj2* and its chimeric genes was performed using hairy root transformation as described by Sugawara et al. [24]. We used binary plasmids pUB-GW-GFP-Rj2 and pUB-GW-GFP-rj2 [24], respectively, for Rj2-WT and Rj2-E452K/I490R complementation. Binary plasmids for Rj2-E452K and Rj2-I490R were constructed as follows. To generate mutated cDNA fragments, G1354 or T1469 (corresponding to residues E452 and I490 in the protein) in *Rj2* cDNA were substituted with A1354 or G1469 by overlap extension PCR, and we used pMS145 (S2 Table) as the template DNA. The DNA fragment obtained was cloned into pENTR/D-TOPO (Thermo Fisher Scientific, Inc.), yielding pMS152 and pMS153. These mutated cDNAs were transferred from the clones into the binary vector pUB-GW-GFP [52] between the polyubiquitin (*LjUbq1*) promoter and the *nos* terminator, using Gateway LR Clonase II (Thermo Fisher Scientific, Inc.). These constructs were used to transfect *A*. *rhizogenes* K599. Six-day-old seedlings of *rj2* soybean cultivar Lee were used for complementation testing; *A*. *rhizogenes*-mediated hairy root transformation was based on the protocol described by Kereszt *et al*. [53]. Briefly, using a syringe, the cotyledonary node was infected with overnight cultures of *A*. *rhizogenes* K599 carrying *Rj2* cDNAs. Infected seedlings were maintained in sterile vermiculite pots in a growth chamber under high humidity until hairy roots developed at the infection site (~2 weeks). These seedlings, with the main roots and non-GFP roots removed under a fluorescence binocular microscope, were cultured for 1 week in sterile vermiculite pots containing half-strength B&D solution [54] and 0.5 mM NH_4_NO_3_. The seedlings were inoculated with *B*. *diazoefficiens* USDA 122 under nitrogen-free conditions [51], and nodulation on transgenic roots was examined 4 weeks after inoculation.

### SNP genotyping of soybean accessions

SNP genotyping was conducted using the MassARRAY system as described by Kaga et al. [7]. Briefly, multiplex PCR with a primer set 1st_PCRP and 2nd_PCRP followed by a template-directed single base extension with a primer UEP_SEQ at the SNP site (*rj2* cDNA sequence position 1470 of soybean cultivar Williams 82) were conducted using the iPLEX Gold kit (Agena Bioscience, Inc. San Diego, CA, USA) following the manufacture’s protocol. The reaction mixture was dispensed onto a silicon matrix preloaded SpectroCHIP (Agena Bioscience, Inc.) using Nanodispenser (Agena Bioscience, Inc.) and analyzed by Compact MassARRAY MALDI-TOF (Agena Bioscience, Inc.). The genotypes were determined using MassARRAY Typer4.0 (Agena Bioscience, Inc.).

### Identification of nucleotide sequences of *Rj2* gene in wild soybean

Total RNA was extracted using a NucleoSpin RNA Plant Kit (Macherey-Nagel Inc., Düren, Germany) and additionally treated with DNase I (Promega Inc., Madison, WI, USA) according to the manufacturer’s instructions. The full-length cDNA sequences of the *Rj2* gene in one of the *G*. *soja* accessions was determined using the 5′ and 3′ rapid amplification of cDNA ends (RACE) technique with the SMARTer RACE 5′/3′ Kit (Takara Bio Inc.). To investigate the *Rj2* cDNA sequences, first-strand cDNA was synthesized from total RNA using a SuperScript III First Strand Synthesis System for RT-PCR (Thermo Fisher Scientific, Inc.) with the gene-specific primer GsRj2_R (S5 Table). The full-length cDNA sequences (3.2 kb) were amplified by PCR using the primer set GsRj2_F and GsRj2_R (S5 Table). The PCR products were purified, and the DNA sequences were determined using the primer walking method. The sequences of the primers used in the analysis are listed in S5 Table.

### Statistical analysis

Statistical significance of formed nodule number between two groups was determined using a two-tailed Student’s t-test performed by Microsoft Excel for pairwise comparisons. For non-normal distribution data from comparisons of multiple test samples, significance of difference among groups was evaluated by Steel–Dwass test using JMP software (SAS Institute Inc. Cary, NC, USA). *P*-values < 0.05 were considered statistically significant. Sample size (*n*) used for experiments is indicated in the figure legends.

## Supporting information

S1 Fig Nodulation phenotype of *Glycine soja* accessions following inoculation with *Bradyrhizobium diazoefficiens* USDA 122 and its derivative carrying USDA 110 -type *nopP* (122*nopP*_110_). (A) Number of nodules formed on roots of *Rj2*-gonotype of *G*. *soja* accessions inoculated with *B*. *diazoefficiens* USDA 122 or 122*nopP*_110_ 28 days after inoculation. Error bars show SEM (*n* = 4). Significant differences from 122*nopP*_110_ were detected using Student’s *t*-test: *P < 0.01. (B) Shoots and roots of accessions JP90948 (*Rj2*) at 28 days after inoculation with *B*. *diazoefficiens* strains. Scale bar: 0.5 mm.
(PDF)

S1 Table *Glycine max* accessions in the Japanese mini-core collection. (DOCX)

S2 Table Bacterial strains and plasmids used in this study. (DOCX)

S3 Table *Glycine soja* accessions used for phenotyping, and the amino acid sequences of their Rj2 or rj2 protein. (DOCX)

S4 Table Soybean accessions used for SNP genotyping. (XLSX)

S5 Table Oligonucleotide primers used in this study. (DOCX)
