# Supplementary material for: Symbiotic incompatibility between soybean and Bradyrhizobium arises from one amino acid determinant in soybean Rj2 protein
Source: PLoS One. 2019 Sep 13;14(9):e0222469. doi: 10.1371/journal.pone.0222469 (PMC6743760; doi:10.1371/journal.pone.0222469)
Supplement: S1 Fig — (A) Number of nodules formed on roots of Rj2-gonotype of G. soja accessions inoculated with B. diazoefficiens USDA 122 or 122nopP110 28 days after inoculation. Error bars show SEM (n = 4). Significant differences from 122nopP110 were detected using Student’s t-test: *P < 0.01. (B) Shoots and roots of accessions JP90948 (Rj2) at 28 days after inoculation with B. diazoefficiens strains. Scale bar: 0.5 mm. (PDF) [file pone.0222469.s001.pdf]

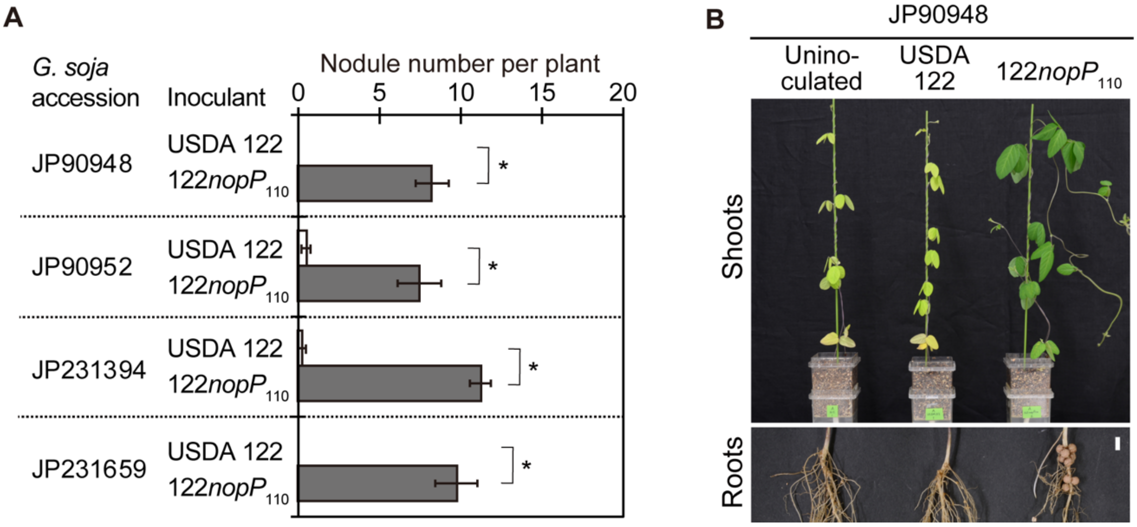

S1 Fig. Nodulation phenotype of *Glycine soja* accessions following inoculation with *Bradyrhizobium diazoefficiens* USDA 122 and its derivative carrying USDA 110-type *nopP* (122*nopP*$_{110}$). (A) Number of nodules formed on roots of *Rj2*-gonotype of *G. soja* accessions inoculated with *B. diazoefficiens* USDA 122 or 122*nopP*$_{110}$ 28 days after inoculation. Error bars show SEM ($n = 4$). Significant differences from 122*nopP*$_{110}$ were detected using Student's *t*-test: *$P < 0.01$. (B) Shoots and roots of accessions JP90948 (*Rj2*) at 28 days after inoculation with *B. diazoefficiens* strains. Scale bar: 0.5 mm.
