# Supplementary material for: Symbiotic incompatibility between soybean and Bradyrhizobium arises from one amino acid determinant in soybean Rj2 protein
Source: PLoS One. 2019 Sep 13;14(9):e0222469. doi: 10.1371/journal.pone.0222469 (PMC6743760; doi:10.1371/journal.pone.0222469)
Supplement: S1 Table — (DOCX) [file pone.0222469.s002.docx]

**S1 Table. *Glycine max* accessions in the Japanese mini-core collection.**

| *G. max* accession ID | Cultivar name | Origin (Prefecture) | Sequence type of Rj2 | GenBank accession of Exon 2 region |
| --- | --- | --- | --- | --- |
| GmJMC013 | CHIZUKA IBARAKI 1 | Ibaraki | JC1 | LC466667 |
| GmJMC041 | DATE CHA MAME | Miyagi | JC1 | LC466680 |
| GmJMC063 | AOBAKO | Yamagata | JC1 | LC466696 |
| GmJMC102 | KURAKAKE | Niigata | JC1 | LC466719 |
| GmJMC105 | MAETSUE ZAIRAI 90B | Oita | JC1 | LC466720 |
| GmJMC158 | KUMAJI 1 | Kumamoto | JC1 | LC466734 |
| GmJMC007 | TOKACHI NAGAHA | Hokkaido | JC2 | LC466664 |
| GmJMC044 | SHAKKIN NASHI | Gunma | JC2 | LC466682 |
| GmJMC080 | HIME DAIZU | Gifu | JC2 | LC466706 |
| GmJMC101 | COL/EHIME/1983/UTSUNOMIYA 22 | Ehime | JC2 | LC466718 |
| GmJMC121 | KOSA MAME | Tochigi | JC2 | LC466727 |
| GmJMC137 | COL/EHIME/1983/UTSUNOMIYA 37 | Ehime | JC2 | LC466732 |
| GmJMC179 | SAGA ZAIRAI | Saga | JC2 | LC466739 |
| GmJMC184 | BAN KURO DAIZU | Kumamoto | JC2 | LC466741 |
| GmJMC056 | TAMAHOMARE | Nagano | JC3 | LC466689 |
| GmJMC058 | YAHAGI | Aichi | JC3 | LC466691 |
| GmJMC061 | KOMAME | Gunma | JC3 | LC466694 |
| GmJMC067 | HOUJAKU | Nagano | JC3 | LC466699 |
| GmJMC078 | AKASAYA | Nara | JC3 | LC466704 |
| GmJMC106 | HIMESHIRAZU | Chiba | JC3 | LC466721 |
| GmJMC117 | AKISENGOKU | Kumamoto | JC3 | LC466726 |
| GmJMC004 | KITAJIRO | Chiba | JC4 | LC466663 |
| GmJMC016 | JUKKOKU | Saitama | JC4 | LC466668 |
| GmJMC021 | OOYACHI 2 | Hokkaido | JC4 | LC466669 |
| GmJMC026 | ONI HADAKA | Tochigi | JC4 | LC466672 |
| GmJMC030 | KURODAIZU(AO HIGUU CHUU) | Okinawa | JC4 | LC466674 |
| GmJMC043 | TAKIYA | Yamagata | JC4 | LC466681 |
| GmJMC050 | FUKUI SHIRO | Fukui | JC4 | LC466685 |
| GmJMC051 | KURODAIZU(GEIHOKU) | Hiroshima | JC4 | LC466686 |
| GmJMC052 | KISAYA(NATSU) | Kagoshima | JC4 | LC466687 |
| GmJMC059 | SOKOSHIN | Niigata | JC4 | LC466692 |
| GmJMC081 | AKUDEN SHIRAZU | Nagano | JC4 | LC466707 |
| GmJMC096 | NAKAHATA ZAIRAI | Shizuoka | JC4 | LC466714 |
| GmJMC098 | AKA DAIZU | Tokushima | JC4 | LC466715 |
| GmJMC099 | AMAGI ZAIRAI 90D | Fukuoka | JC4 | LC466716 |
| GmJMC130 | HITASHIMAME | Yamagata | JC4 | LC466730 |
| GmJMC149 | MOCHI-DAIZU | Mie | JC4 | LC466733 |
| GmJMC161 | ITSUKI ZAIRAI 83H | Kumamoto | JC4 | LC466735 |
| GmJMC172 | TSURUSENGOKU | Chiba | JC4 | LC466737 |
| GmJMC008 | KANAGAWA WASE | Kumamoto | JC5 | LC466665 |
| GmJMC032 | NATTOU KOTSUBU | Ibaraki | JC6 | LC466675 |
| GmJMC076 | IHHON SANGOU | Ibaraki | JC7 | LC466702 |
| GmJMC167 | NANKAN ZAIRAI 83 | Kumamoto | JC7 | LC466736 |
| GmJMC180 | KOMUTA | Kumamoto | JC8 | LC466740 |
| GmJMC009 | SHIZUNAIDAIZU | Hokkaido | JC9 | LC466666 |
| GmJMC023 | KUROGOYOU | Fukushima | JC9 | LC466670 |
| GmJMC025 | ENREI | Nagano | JC9 | LC466671 |
| GmJMC028 | KOITO | Chiba | JC9 | LC466673 |
| GmJMC034 | MIYAGI SHIROME | Miyagi | JC9 | LC466676 |
| GmJMC037 | YAKUMO MEAKA | Hokkaido | JC9 | LC466677 |
| GmJMC039 | NATTOUMAME | Nagano | JC9 | LC466678 |
| GmJMC040 | KOIBUCHIMURA ZAIRAI | Ibaraki | JC9 | LC466679 |
| GmJMC047 | AKITA ANI | Yamagata | JC9 | LC466683 |
| GmJMC049 | HIKU ANDA | Okinawa | JC9 | LC466684 |
| GmJMC053 | ABURA MAME | Fukushima | JC9 | LC466688 |
| GmJMC057 | SHAKUJOU MAME | unknown | JC9 | LC466690 |
| GmJMC060 | SHIMO HISAKATA DAIZU | Nagano | JC9 | LC466693 |
| GmJMC062 | AZEMAME | Tochigi | JC9 | LC466695 |
| GmJMC064 | MEGURO 1 | Aomori | JC9 | LC466697 |
| GmJMC065 | OOJIRO | Gunma | JC9 | LC466698 |
| GmJMC068 | ZAIRAI 51-2 | Aichi | JC9 | LC466700 |
| GmJMC069 | CHADAIZU | Miyagi | JC9 | LC466701 |
| GmJMC077 | HITORIMUSUME | Yamagata | JC9 | LC466703 |
| GmJMC079 | KURUMIMAME | Miyagi | JC9 | LC466705 |
| GmJMC082 | AOAKIMAME | Hyogo | JC9 | LC466708 |
| GmJMC085 | DAIZU | Wakayama | JC9 | LC466709 |
| GmJMC088 | CHUU TEPPOU | Gifu | JC9 | LC466710 |
| GmJMC090 | DADACHAMAME | Yamagata | JC9 | LC466711 |
| GmJMC091 | KUROTOME | Miyagi | JC9 | LC466712 |
| GmJMC092 | KUROHIRA | Iwate | JC9 | LC466713 |
| GmJMC100 | KUROMAME | Saitama | JC9 | LC466717 |
| GmJMC110 | COL/TANBA/1989/ODAGAKI 2 | Hyogo | JC9 | LC466722 |
| GmJMC112 | FUKUYUTAKA | Kumamoto | JC9 | LC466723 |
| GmJMC114 | COL/EHIME/1-2 | Ehime | JC9 | LC466724 |
| GmJMC116 | SHIRATAMA | Iwate | JC9 | LC466725 |
| GmJMC126 | KOKUBU 7 | Hyogo | JC9 | LC466728 |
| GmJMC128 | GIN DAIZU | Okayama | JC9 | LC466729 |
| GmJMC131 | COL/EHIME/1983/UTSUNOMIYA 28 | Ehime | JC9 | LC466731 |
| GmJMC177 | HAI MAME | Yamanashi | JC9 | LC466738 |
