# Supplementary material for: Symbiotic incompatibility between soybean and Bradyrhizobium arises from one amino acid determinant in soybean Rj2 protein
Source: PLoS One. 2019 Sep 13;14(9):e0222469. doi: 10.1371/journal.pone.0222469 (PMC6743760; doi:10.1371/journal.pone.0222469)
Supplement: S2 Table — (DOCX) [file pone.0222469.s003.docx]

**S2 Table. Bacterial strains and plasmids used in this study.**

| Strain or plasmid | Relevant characteristic(s) | Reference or source |
| --- | --- | --- |
| *Bradyrhizobium diazoefficiens* strains | | |
| USDA 122 | Wild type, incompatible with *Rj2* soybean; Px^r^ | USDA |
| 122Ω*rhcJ* | USDA 122 *rhcJ*::Ω cassette; Px^r^; Sp^r^; Sm^r^ | Tsukui et al. 2013 |
| 122*nopP*_110_ | USDA 122 exchanged *nopP* to USDA 110-type gene, Px^r^ | Sugawara et al. 2018 |
| USDA 110^T^ | Wild type, compatible with *Rj2* soybean; Px^r^ | USDA |
| *Agrobacterium rhizogenes* strain | | |
| K599 | Cucumopine-type for induction of hairy root transformation | Savka et al. 1990 |
| *Escherichia coli* strains | |  |
| DH5α | F－, Φ80d *lacZ*ΔM15, Δ(l*acZYA*-*argF*)U169, *deoR, recA1, endA1*, *hsdR17*(rK- mK+), *phoA*, *supE44*, λ－, *thi*-1, *gyrA96*, *relA1* | Nippon Gene Inc. |
|  |  |  |
| Plasmids | |  |
| pENTR/dTOPO | Cloning vector; Km^r^ | Thermo Fisher Scientific Inc. |
| pUB-GW-GFP | Binary vector for *Agrobacterium*-mediated hairy root transformation; Km^r^ | Maekawa et al., 2008 |
| pMS145 | pENTR/dTOPO containing *Rj2* cDNA from *G. max* cv. Hardee; Km^r^ | Sugawara et al. 2018 |
| pMS152 | pENTR/dTOPO containing *Rj2* cDNA substituted to A1354 | This study |
| pMS153 | pENTR/dTOPO containing *Rj2* cDNA substituted to G1469 from *G. max* cv. Hardee; Km^r^ | This study |
| pUB-GW-GFP-*Rj2* | pUB-GW-GFP containing *Rj2* cDNA downstream of the *LjUbq1* promoter; Km^r^; Cm^r^ | Sugawara et al. 2018 |
| pUB-GW-GFP-*Rj2*-E452K | pUB-GW-GFP containing *Rj2* cDNA substituted to A1354 at downstream of *LjUbq1* promoter; Km^r^; Cm^r^ | This study |
| pUB-GW-GFP-*Rj2*-I490R | pUB-GW-GFP containing *Rj2* cDNA substituted to G1469 at downstream of *LjUbq1* promoter; Km^r^; Cm^r^ | This study |
| pUB-GW-GFP-*rj2* | pUB-GW-GFP containing Hardee *Rj2* cDNA substituted to A1354 and G1469 at downstream of *LjUbq1* promoter; Km^r^; Cm^r^ | Sugawara et al. 2018 |

USDA, U.S. Department of Agriculture, Beltsville, MD. Km^r^, kanamycin resistant; Px^r^, polymyxin B resistant; Sp^r^, spectinomycin resistant, Sm^r^, streptomycin resistant, Cm^r^, chloramphenicol resistant.

References for Table S2:

Maekawa et al. 2008. Polyubiquitin Promoter-Based Binary Vectors for Overexpression and Gene Silencing in *Lotus japonicus*. Mol. Plant-Microbe Interact. 21:375–382

Savka 2007. Induction of Hairy Roots on Cultivated Soybean Genotypes and Their Use to Propagate the Soybean Cyst Nematode. Phytopathology. 80:503

Sugawara et al. 2018. Variation in bradyrhizobial NopP effector determines symbiotic incompatibility with *Rj2*-soybeans via effector-triggered immunity. Nat. Commun. 9:3139

Tsukui et al. 2013. The Type III Secretion System of *Bradyrhizobium japonicum* USDA122 Mediates Symbiotic Incompatibility with *Rj2* Soybean Plants. Appl. Environ. Microbiol. 79:1048–1051
