# Supplementary material for: Symbiotic incompatibility between soybean and Bradyrhizobium arises from one amino acid determinant in soybean Rj2 protein
Source: PLoS One. 2019 Sep 13;14(9):e0222469. doi: 10.1371/journal.pone.0222469 (PMC6743760; doi:10.1371/journal.pone.0222469)
Supplement: S3 Table — (DOCX) [file pone.0222469.s004.docx]

**S3 Table. *Glycine soja* accessions used for phenotyping, and the amino acid sequences of their Rj2 or rj2 protein.**

| *G. soja* accession ID | Origin | Genotype*^a^* | Number of amino acids | Sequence identity with Hardee Rj2*^b^* | GenBank Accession |
| --- | --- | --- | --- | --- | --- |
| JP90948 | Japan, Fukui | *Rj2* | 1052 | 1035/1052 (98%) | LC466656 |
| JP90952 | Japan, Hyogo | *Rj2* | 1052 | 1038/1052 (98%) | LC466657 |
| JP231394 | Korea, Kyongsang | *Rj2* | 1052 | 1039/1052 (98%) | LC466658 |
| JP231659 | Korea, Kyongsang | *Rj2* | 1052 | 1050/1052 (99%) | LC466659 |
| JP110740 | Japan, Kumamoto | *rj2* | 1070 | 1030/1072 (96%) | LC466660 |
| JP233152*^c^* | Japan, Kyoto | *rj2* | 931 | 883/929 (95%) | LC466662 |
| JP231372 | Korea, Kangwong | *rj2* | 1052 | 1045/1052 (99%) | LC466661 |
| JP231484*^d^* | Korea, Chungchong | *rj2* | - | - | - |

*a*: Genotypes determined using SNP genotyping for I490; nodulation phenotype inoculated with *B. diazoefficiens* USDA 122.

*b*: Amino acid sequence of Rj2 protein in *G. max* cv. Hardee (1052 aa, Genbank accession no. ADF78112) used for comparisons.

*c*: Truncated protein was deduced because there was a single nucleotide deletion in the C-terminal of the cDNA.

*d*: The Rj2 cDNA failed to amplify from the RNA of this accession.
