# Supplementary material for: Symbiotic incompatibility between soybean and Bradyrhizobium arises from one amino acid determinant in soybean Rj2 protein
Source: PLoS One. 2019 Sep 13;14(9):e0222469. doi: 10.1371/journal.pone.0222469 (PMC6743760; doi:10.1371/journal.pone.0222469)
Supplement: S5 Table — (DOCX) [file pone.0222469.s006.docx]

**S5 Table. Oligonucleotide primers used in this study.**

| Name | Sequence (5'→3′) | Purpose |
| --- | --- | --- |
| Glyma_Rgene_F2 | TGTAAAGAGAAAAGGCCGAGAG | Amplification and sequencing of Exon 2 of *Rj2/rj2* in soybean |
| Glyma_Rgene_R2 | ACAACCACAATGCAAGTAAACG | Amplification and sequencing of Exon 2 of *Rj2/rj2* in soybean |
| Rj2_Y2H_F2 | CACCATGGCTTTGGGATCATGTTCCTC | Cloning of *Rj2* cDNA of Hardee |
| Rj2_Y2H_R3 | CTACATCATCGTCTGATTTTCTCTTGC | Cloning of *Rj2* cDNA of Hardee |
| K452_F | TGACTTGACAAAGGTTGAAGAT | Cloning of mutated *Rj2* cDNA by overlap extension PCR |
| K452_R | ATCTTCAACCTTTGTCAAGTCA | Cloning of mutated *Rj2* cDNA by overlap extension PCR |
| R490_F | GTAGAGTACCTAGAGTTACAATGC | Cloning of mutated *Rj2* cDNA by overlap extension PCR |
| R490_R | GCATTGTAACTCTAGGTACTCTAC | Cloning of mutated *Rj2* cDNA by overlap extension PCR |
| Rj2_3-RACE | GATTACGCCAAGCTTSAATGCATGACTTGATTSAGGACATGRG | 3′-RACE for *GsRj2* |
| Rj2_5-RACE | GATTACGCCAAGCTTCYCATGTCCTSAATCAAGTCATGCATTS | 5′-RACE for *GsRj2* |
| M13-47 | CGCCAGGGTTTTCCCAGTCACGAC | 5′/3′-RACE and *Rj2* sequencing |
| 1st_PCRP | ACGTTGGATGTTCGGTGATTCTTGCCTGAC | SNP genotyping for I490 |
| 2nd_PCRP | ACGTTGGATGGTTTAGTTGGTATGGTAGAG | SNP genotyping for I490 |
| UEP_SEQ | TGGTATGGTAGAGTACCTA | SNP genotyping for I490 (for single base extension) |
| GsRj2_F | AGTAGACTTTCTTCTCAGTTCAC | Amplification and sequencing of *Rj2/rj2* in wild soybean |
| GsRj2_R | CATACAATTTCCACTGCCCAG | Amplification and sequencing of *Rj2/rj2* in wild soybean |
| GsRj2_F2 | CATGGAATTGGTGGGATAGGA | Sequencing of *Rj2/rj2* |
| GsRj2_R2 | CTTGCTGGAGACCAACTCAAC | Sequencing of *Rj2/rj2* |
| GsRj2_R3 | AATTGGGATCGAATGGGTGC | Sequencing of *Rj2/rj2* |
| Rj2_F1 | GTTGAGTTGGTCTCCAGCAAG | Sequencing of *Rj2/rj2* |
| Rj2_F2 | CCTTGACATTGCCTGTTGC | Sequencing of *Rj2/rj2* |
| Rj2_F3 | GCTGGGCTTCAAGCATTAG | Sequencing of *Rj2/rj2* |
| Rj2_F4 | GATAGGTTCCAACTTGTTCGGG | Sequencing of *Rj2/rj2* |
| Rj2_F5 | TGAGAGTACTGGAATGGTGGAG | Sequencing of *Rj2/rj2* |
| Rj2_R1 | CCTTTCCTGATGCTTAGCCA | Sequencing of *Rj2/rj2* |
| Rj2_R2 | CCCGAACAAGTTGGAACC | Sequencing of *Rj2/rj2* |
| Rj2_R3 | GTCAGTTCCGGCATCAGGAC | Sequencing of *Rj2/rj2* |
| Rj2_R4 | TGTCAAAGCTGGGGAAGTCT | Sequencing of *Rj2/rj2* |
